# Spatially explicit assessment of heat health risk by using multi-sensor remote sensing images and socioeconomic data in Yangtze River Delta, China

**DOI:** 10.1186/s12942-018-0135-y

**Published:** 2018-05-25

**Authors:** Qian Chen, Mingjun Ding, Xuchao Yang, Kejia Hu, Jiaguo Qi

**Affiliations:** 1 Institute of Island and Coastal Ecosystems, Ocean College, Zhejiang University, Zhoushan, 316021 China; 2 Key Lab of Poyang Lake Wetland and Watershed Research of Ministry of Education, School of Geography and Environment, Jiangxi Normal University, Nanchang, 330022 China; 3 Center for Global Change and Earth Observations, Michigan State University, East Lansing, MI 48823 USA

**Keywords:** Spatial risk assessment, Heat health risk, Remote sensing, GIS, Yangtze River Delta

## Abstract

**Background:**

The increase in the frequency and intensity of extreme heat events, which are potentially associated with climate change in the near future, highlights the importance of heat health risk assessment, a significant reference for heat-related death reduction and intervention. However, a spatiotemporal mismatch exists between gridded heat hazard and human exposure in risk assessment, which hinders the identification of high-risk areas at finer scales.

**Methods:**

A human settlement index integrated by nighttime light images, enhanced vegetation index, and digital elevation model data was utilized to assess the human exposure at high spatial resolution. Heat hazard and vulnerability index were generated by land surface temperature and demographic and socioeconomic census data, respectively. Spatially explicit assessment of heat health risk and its driving factors was conducted in the Yangtze River Delta (YRD), east China at 250 m pixel level.

**Results:**

High-risk areas were mainly distributed in the urbanized areas of YRD, which were mostly driven by high human exposure and heat hazard index. In some less-urbanized cities and suburban and rural areas of mega-cities, the heat health risks are in second priority. The risks in some less-developed areas were high despite the low human exposure index because of high heat hazard and vulnerability index.

**Conclusions:**

This study illustrated a methodology for identifying high-risk areas by combining freely available multi-source data. Highly urbanized areas were considered hotspots of high heat health risks, which were largely driven by the increasing urban heat island effects and population density in urban areas. Repercussions of overheating were weakened due to the low social vulnerability in some central areas benefitting from the low proportion of sensitive population or the high level of socioeconomic development. By contrast, high social vulnerability intensifies heat health risks in some less-urbanized cities and suburban areas of mega-cities.

## Background

Climate change, with global warming as the main feature, has become the biggest challenge for human health [1, 2]. Extreme heat events (EHEs), as one of the most serious meteorological disasters, are projected to increase in frequency, intensity, and duration in the background of future climate warming [3]. Exposure to high ambient temperature not only is the leading cause of weather-related morbidity (e.g., cardiovascular, cerebrovascular, and respiratory diseases) [4] but may also lead to human deaths in extreme cases. For example, two devastating heat wave in Europe in 2003 and Russia in 2010 led to a death toll of 70,000 and 55,000, respectively [5, 6]. The health implications of extreme heat events highlight the significance of studies on risk assessment and identification of high-risk population. Climate change adaptation is also increasingly linked to natural hazard risk management [7, 8].

There are ongoing observational studies that show a significant increase in the incidence and mortality of urban residents during EHEs [9, 10], which are associated with the urban heat island (UHI) whose effects are aggravated during heat waves [4, 11, 12]. Dousset et al. [13] analyzed mortality in Paris during EHEs in the summer of 2003; results showed that mortality is related to the distribution of high nighttime temperatures generally driven by the enhanced UHI effects at night. Urban residents are therefore particularly vulnerable to severe and sustained heat stress [14, 15]. Spatially explicit identification of high-risk hotspots will ensure appropriate development of targeted prevention and mitigation of EHEs in a warmer future world considering the projected augmentation in urban population and frequency of EHEs.

An increasing number of studies utilized the risk conceptual framework proposed by Crichton [8, 16] to fully understand heat-related risk patterns. However, there usually exist two deficiencies in previous heat health risk assessment, which related to heat hazard and human exposure respectively. For heat-related health risk, hazards refer to the possibility of EHEs occurring in a specific space where people live or engage in anthropogenic activities, characterizing the closeness of humans to EHEs [17, 18]. For a large study area, available air temperature data are commonly from the sparse government-operated stations. Those existing data are constrained by their spatial locations and are therefore inadequate for capturing the temperature gradient within a specific area. Previous studies on the association between ambient temperature and mortality also pointed out that the use of temperature data from sparse weather stations led to underestimation of the temperature effects [19]. In addition to coarse heat hazard information obtained from meteorological station [20, 21], satellite-derived land surface temperature (LST) data were increasingly used to measure heat risk because they offer spatially-detailed heat-related information [22–25]. Moreover, it is noteworthy that although the synergies between UHI and heat waves have received increasing attention because of their potential health and environmental impacts [11, 26], most studies on spatial heat hazard assessment only considered the daytime temperature but ignored the UHI-related nighttime temperature [23, 27], which may result in significant underestimation of heat health risk in urban areas.

For human exposure analysis, demographic data is a fundamental component of disaster risk models. Detailed population information is required to assess casualties, determine shelter needs, and properly implement evacuation plans in pre-disaster and post-disaster phases [28, 29]. The absence of population data is a major obstacle to decision-making and disaster relief in parts of the developing world due to the lack of data collection or the unavailability of useful accompanying geographical data [30]. Population density maps on the basis of census data lack sufficient spatial details of the geographically-heterogeneous population distribution within the border of the census units, leading to a spatial mismatch with spatially explicit hazard data in risk assessment [17]. Emerging geospatial technologies, such as remote sensing and geographical information systems (GIS) techniques, are powerful tools for estimating population density at a finer scale. The GIS-based integration of multi-source remote sensing images can serve as a proxy for spatially explicit assessment of human exposure [24]. Therefore, the widely available datasets and the flexibility of GIS techniques make it possible to develop an effective and low-cost method for identifying the high exposure hotspots at finer scales, even for developing countries.

Currently, most studies on heat health risk assessment have been conducted in developed countries and mainly focused on cities. It is noteworthy that the spatial distribution of heat risk in developing countries is generally less well known [15, 31]. Furthermore, existing studies have been mainly implemented at the administrative unit level while few attempts focus on the specializing of heat-related health risk at a raster level. In this study, we aim to assess heat-related health risk at regional scale and explore its driving factors at a high spatial resolution. Herein, we took the Yangtze River Delta (YRD) in east China as a case study. A composite heat risk index aggregating three risk elements (heat hazard, human exposure, and vulnerability) was generated to improve the spatial delineation of heat health risk by comprehensive utilization of multi-source data. The spatially explicit heat health risk map and its driving factors were explored at the 250 m pixel level across the YRD, which can provide scientific foundation for effective resource targeting and beneficial program interventions with the least field-collection efforts.

## Methods

### Study area

**Fig. 1:**
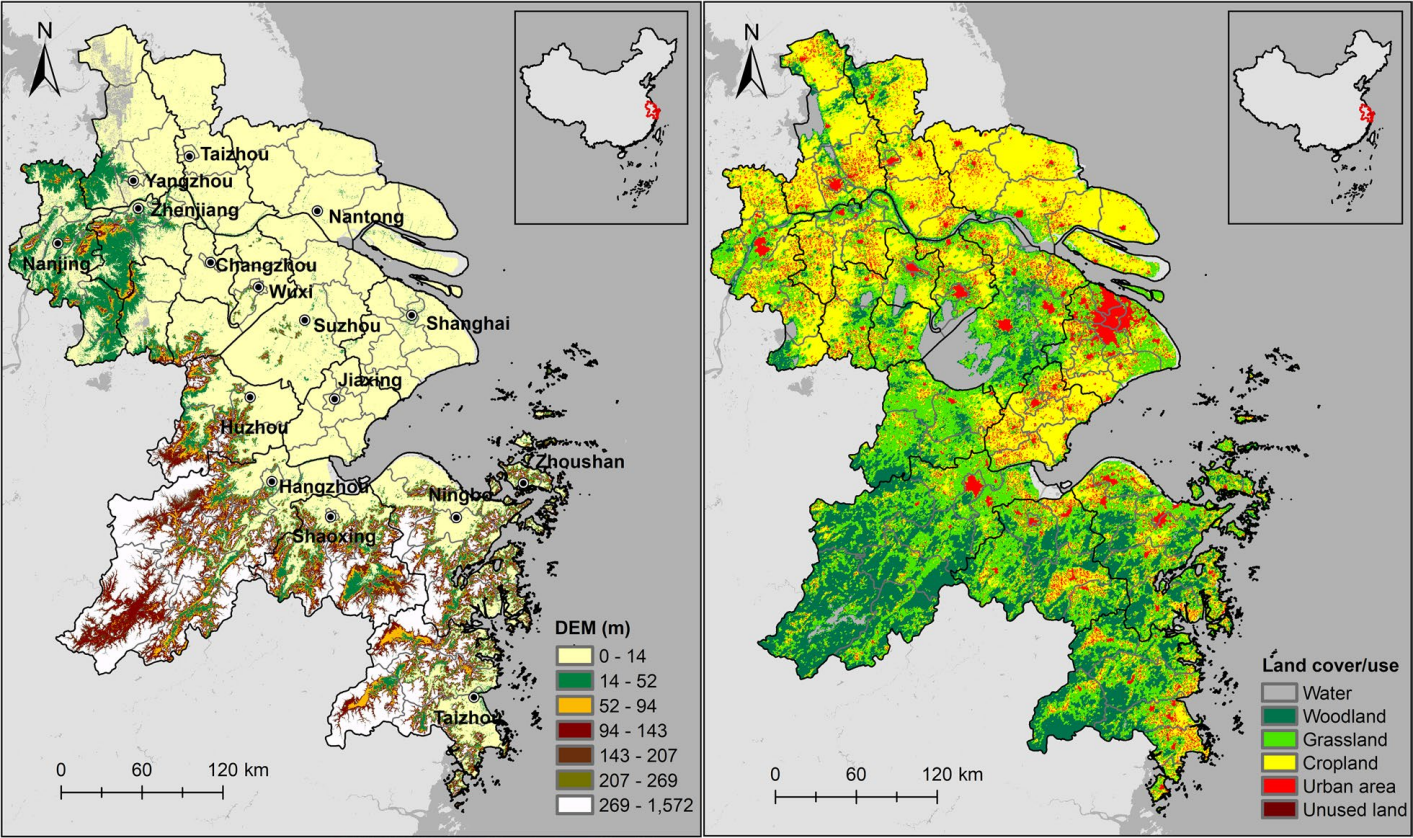
Study area location, elevation, and land cover types

The YRD lies along the eastern coast of China, including Shanghai, Hangzhou, Ningbo, Jiaxing, Shaoxing, Zhoushan, Huzhou, Taizhou, Nanjing, Suzhou, Yangzhou, Changzhou, Nantong, Wuxi, Zhenjiang, and Taizhou cities that are highly prosperous and populous (Fig. 1). The region is bounded by 116.78°E to 124.21°E and 26.99°N to 34.64°N and spread around 112,642 km^2^. The YRD is located in the subtropical monsoon climate zone with a humid monsoon climate. Its annual average temperature ranges from 18 to 23 °C, and the average annual rainfall is approximately 1500 mm. During summer the YRD is frequently threatened by EHEs due to the long-lasting impact of the west Pacific subtropical high [32]. In July and August, there are usually a total of 20–30 hot days (daily maximum temperature ≥ 35 °C), with more than 40 hot days in some specific years. For example, in the summer of 2013, air temperature observations and the number of hot days in many cities of the YRD broke the historical records of the last 50 years [33]. The numbers of hot days were 47, 53, and 37 for Shanghai, Hangzhou, and Nanjing, respectively. In addition, the YRD has experienced unprecedented economic development and urban expansion in the past 4 decades [34], which has resulted in the intensified UHI effect and a large increase in the heat-related health risk [35, 36].

### Data collection and pre-processing

#### Satellite data

1. Temperature data. LST data based on moderate-resolution imaging spectroradiometer (MODIS) on board the National Aeronautics and Space Administration (NASA) EOS Terra and Aqua satellites are distributed as the MOD11A1 (daytime) and MYD11A1 (nighttime) products [37]. The MODIS LST images include daytime and nighttime measurements with spatial resolution of 1 km. In this study, we chose LST data from an exceptional hot day of August 7, 2013, with maximum air temperature exceeding 40 °C in many cities of YRD. Two clear sky LST images acquired at 10:30 a.m. and 1:30 a.m. were used. The MODIS reprojection tool was used for the mosaicking, reprojection, and resampling of original images, and the new LST images were generated with Albers conical equal area projection at the resolution of 250 m.
2. Vegetation Index data. The MODIS enhanced vegetation index (EVI) dataset (MOD13Q1) in 2013 was freely downloaded from the NASA website at a resolution of 250 m [37]. In comparison with the normalized differential vegetation index, EVI was produced by further minimization of the atmospheric effects and background spectral signals and was more sensitive to high biomass regions. To further eliminate cloud contamination and other noises, maximum value composite method was employed on the multi-temporal MODIS EVI dataset to generate a new EVI composite (${\text{EVI}}_{\max}$), as expressed in Eq. (1):
  1 $${\text{EVI}}_{\max} = {\text{MAX}}\left( {{\text{EVI}}_{1} ,{\text{EVI}}_{2} , \ldots ,{\text{EVI}}_{23} } \right),$$
  where ${\text{EVI}}_{1} ,{\text{EVI}}_{2} , \ldots ,{\text{EVI}}_{23}$ are the original 16 d EVI images in the study area in 2013. Then, the MODIS reprojection tool was utilized for data mosaicking. The new ${\text{EVI}}_{\max}$ image was re-projected into the Albers conical equal area projection.
3. Nighttime light data. The Defense Meteorological Satellite Program’s Operational Linescan System (DMSP/OLS) images can monitor the lights associated with nighttime human activities. Since 1992, the National Geophysical Data Center annually releases global stable DMSP/OLS nighttime light composites that eliminated the cloud, accidental fire, and other noises with a spatial resolution of 30 arc-second [38]. The digital number (DN) values of DMSP/OLS data ranging from 0 to 63 and high DN values in the images generally indicate highly concentrated human activities or settlements. In this study, the original DMSP/OLS data for the year 2012 were projected and resampled to a new raster with Albers conical equal area projection at a resolution of 250 m.
4. Digital elevation model (DEM) data. The elevation data used in this study were downloaded from the website of ERSDAC of Japan [39], comprised the ASTER GDEM (Advanced Spaceborne Thermal Emission and Reflection Radiometer Global Digital Elevation Model) Version 2 with a spatial resolution of 30 m. The original DEM data were re-projected into Albers conical equal area projection and resampled to a new image at a spatial resolution of 250 m to match other datasets.

#### Census data

Census-aged population data at the county level of the study area were derived from China’s Sixth National Census in 2010. Other demographic and socioeconomic statistical data were obtained from the statistical yearbooks of Shanghai, Zhejiang, and Jiangsu provinces and from some local bureaus of statistics for the year 2013.

### Heat health risk assessment framework

The characteristics of natural disasters and their impact depend not only on the frequency or intensity but also on the human exposure and vulnerability [40]. We utilized a spatial heat health risk assessment framework based on the Crichton’s risk triangle [16], which described risk as a function of hazard, human exposure, and vulnerability. For EHEs, heat hazard increased with enhanced temperature and presented a spatial gradient. This enhancement in temperature and resulting heat hazard index was measured using the satellite measured LST data across the YRD. A human settlement index integrated by multi-source data was used to obtain the gridded human exposure index, matching the hazard layer at the spatial scale. For heat vulnerability assessment, multiple demographic and socioeconomic indicators have been reported to associate with hot weather mortality in previous studies [41–43]. Based on literature review [44–47] and data availability, six indicators were chosen to construct a heat vulnerability index. We selected significant components through principal component analysis (PCA) and derived their spatial distribution. The normalized heat hazard index, human exposure index, and heat vulnerability index were multiplied by equal weights to develop a final heat risk index layer given that standard conclusion on the determination of weightings among each indicator in the current risk assessment did not exist [20, 41].

### Hazard

Although the remote-sensed LST that describes the radiometric surface temperature cannot directly represent air temperature, many studies have shown the strong correlation between these two disparate data, particularly at night [48, 49]. Satellite thermal data are therefore increasingly employed to estimate heat hazard [22–24, 27]. The MODIS LST data were selected in this case for their increased spatial coverage, daily measurement, and thermal accuracy, which make it possible to capture complex intra-urban gradient of surface temperature across the study area. Two clear sky LST images for daytime and nighttime during an exceptional heat wave were utilized for heat hazard analysis, considering the quality of LST image and cloud contamination in the study area [50]. Very few no-data pixels were replaced by the mean value of the surrounding 3 × 3 pixel. Then, two LST images were simply added and normalized to obtain the heat hazard index in the study area ranging from 0 to 1 using the ArcGIS software.

### Exposure

The DMSP/OLS image was widely used as a valuable covariate for population density estimation across the world [51, 52]. However, the application of this method is limited by the spatial resolution, overglow, and saturation effects [53, 54]. By combining the vegetation indices (e.g. NDVI) and DMSP/OLS data, the saturation effect in DMSP/OLS data can be greatly reduced [55, 56]. By further incorporating elevation information, Yang et al. [57] proposed an elevation-adjusted human settlement index (EAHSI) at 250 m resolution that can reduce errors in the population estimation among areas with complex terrain. On the basis of the method proposed by Yang et al., an EAHSI at 250 m resolution was obtained in this study by combining DMSP/OLS night light images, EVI, and DEM data using the following formula:

2 $${\text{EAHSI}} = \frac{{\left( {1 - {\text{EVI}}_{\max} } \right) + {\text{OLS}}_{\text{nor}} }}{{1 - {\text{OLS}}_{\text{nor}} + {\text{EVI}}_{\max} + {\text{OLS}}_{\text{nor}} \times {\text{EVI}}_{\max} }} \times {\text{e}}^{{ - 0.003{\text{DEM}}}} ,$$

where

3 $${\text{OLS}}_{\text{nor}} = \left( {{\text{OLS}} - {\text{OLS}}_{\min} } \right)/\left( {{\text{OLS}}_{\max} - {\text{OLS}}_{\min} } \right).$$

The ${\text{OLS}}_{\text{nor}}$ is the normalized value of DMSP/OLS DN image, while ${\text{OLS}}_{\max}$ and ${\text{OLS}}_{\min}$ are the maximum and minimum DN values across the study area, respectively. Correlation analysis between census population and EAHSI in the next section suggested a highly linear relationship (Fig. 4). Then, EAHSI was normalized to generate a heat exposure index ranging from 0 to 1 to characterize the human exposure of EHEs.

### Vulnerability

Many studies suggested that the elderly are more sensitive to EHEs because of their relatively special physiological characteristics and low tolerance to high temperatures [45]. More pressingly, elderly who live alone experience difficulty in obtaining quick and effective aid under emergency conditions [58], exposing them to the considerable threat of EHEs [59]. Meanwhile, individual or regional socioeconomic status plays a role in reducing the vulnerability of related populations. Higher socioeconomic status implies lower heat-related mortality [47]. Air conditioners are considered powerful tools to alleviate the hazardous effects of high temperature [46, 58]. The occupant’s educational background and knowledge of environmental risks affect the individual’s cognitive ability and avoidance behavior to EHEs [20, 60], while the regional economic level and accessibility to medical resources and facilities generally determine human adaptability to EHEs [15].

Based on the review of existing literature and the data availability in the study area, six vulnerability variables were obtained at county level, including age (≥ 65), the elderly who live alone (≥ 60), illiteracy or semi-illiteracy rates of population aged ≥ 15, total beds of health institutions, number of air-conditioning units per 100 households, and per capita GDP. PCA is the primary statistical procedure for constructing social vulnerability index following the methodology by Cutter et al. [61]. PCA could provide information about the spatial structure of the data [62], which enables a few independent components to capture the multi-dimensionality of social vulnerability on the basis of underlying relationships between variables [63]. In this study, PCA was performed to a set of census variables with SPSS software, and the groups of variables with similar spatial patterns were identified as principal components. Once the principal components were produced, the heat vulnerability index was created by summing all the principal components using equal weighting according to their positive (+) or negative (−) effect on vulnerability. Finally, the heat vulnerability index was re-normalized to [0, 1] and mapped at the county level.

## Results

### Heat hazard

**Fig. 2:**
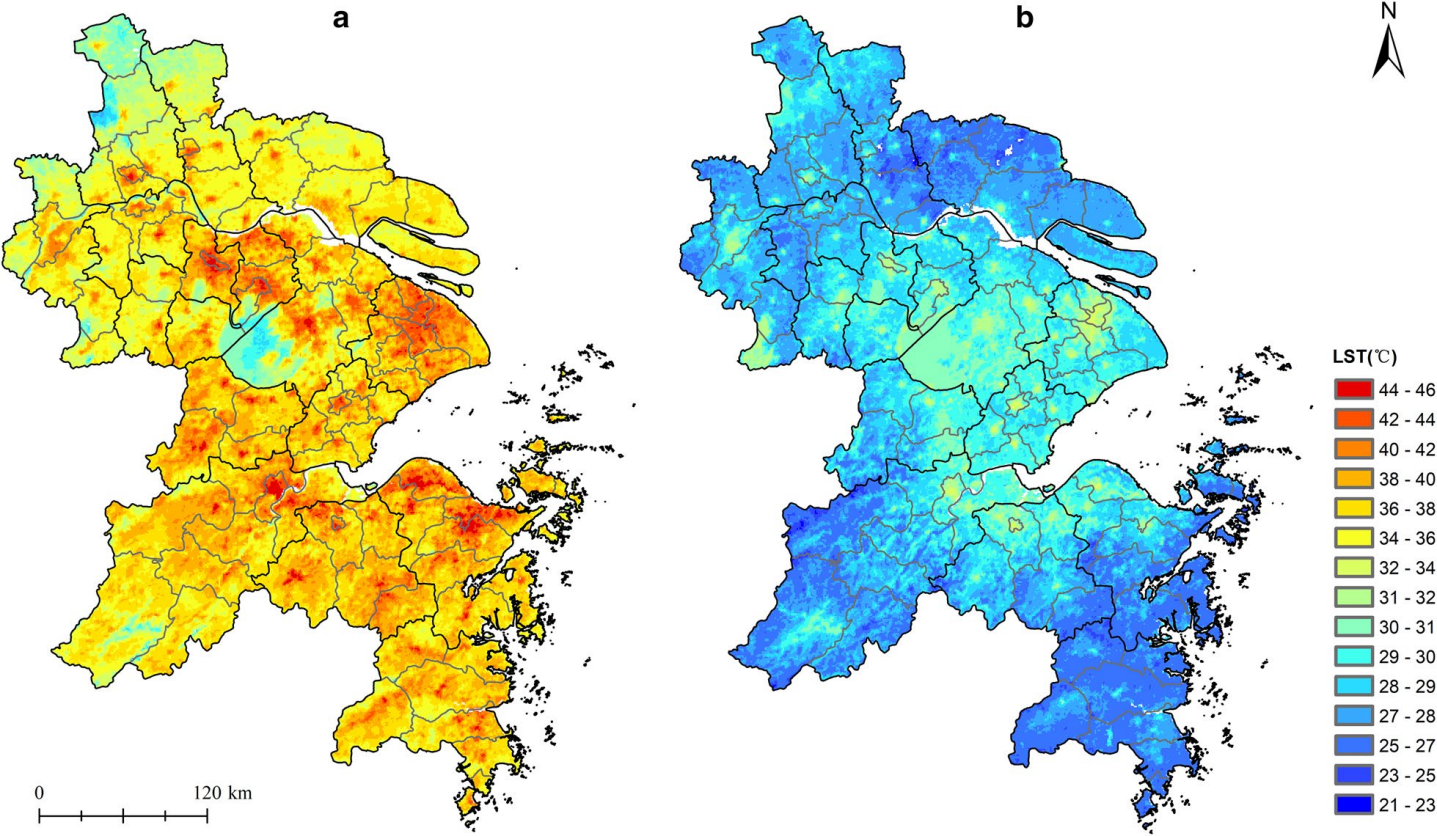
**a** Daytime land surface temperature (LST) and **b** nighttime LST in the Yangtze River Delta

**Fig. 3:**
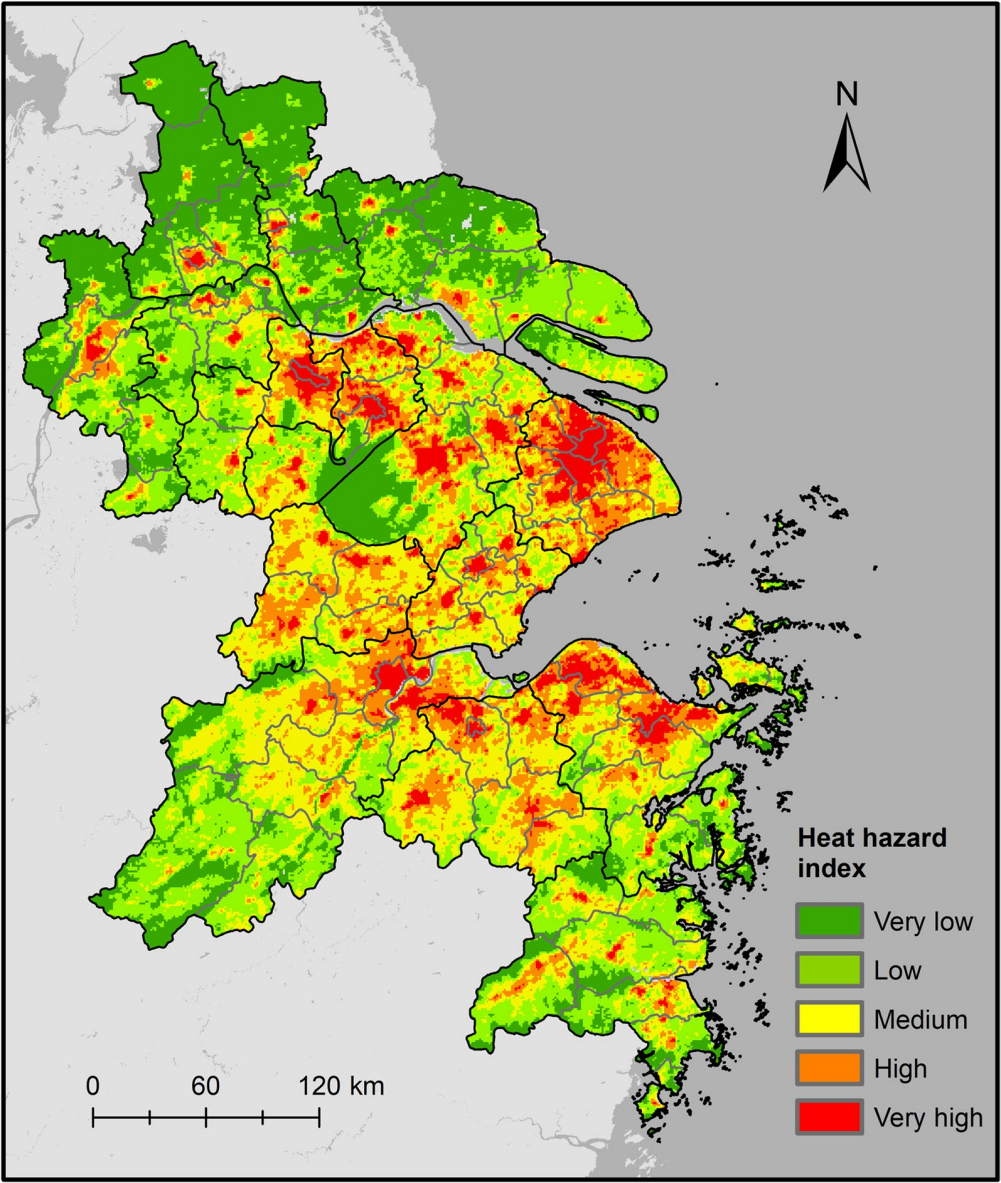
Map of the heat hazard index of the Yangtze River Delta

As shown in Fig. 2, strong and heterogeneous UHI effects for both daytime and nighttime were apparent in the YRD under heat wave conditions. During daytime, the LST varied from 27 to 48 °C, which depicted obvious spatial temperature gradient (Fig. 2a). The daytime hotspots were mainly distributed in the Z-shaped urban agglomeration in YRD, including Changzhou, Wuxi, Suzhou, Shanghai, Hangzhou, Shaoxing, and Ningbo (Fig. 2a). The LST was generally above 40 °C and reached a maximum of 45 °C in the downtowns of Hangzhou and Ningbo. Low daytime temperatures were observed in northern YRD and areas covered by forest and water bodies (such as Taihu Lake with LST ≤ 30 °C). During nighttime, the LST presented weaker temperature gradient across the YRD (Fig. 2b). Nighttime warming centers also concentrated in highly urbanized areas (≥ 30 °C) and expanded to neighboring area, demonstrating a considerable UHI effect at night. The lowest nighttime temperatures were observed in coastal areas and pixels covered by the flourish vegetation in the southern area. The heat hazard index by combining the daytime and nighttime LSTs indicated that the highly-affected areas during EHEs in the YRD are generally concentrated in urban areas, resulting from the coupling effect of the UHI (Fig. 3).

### Exposure

**Fig. 4:**
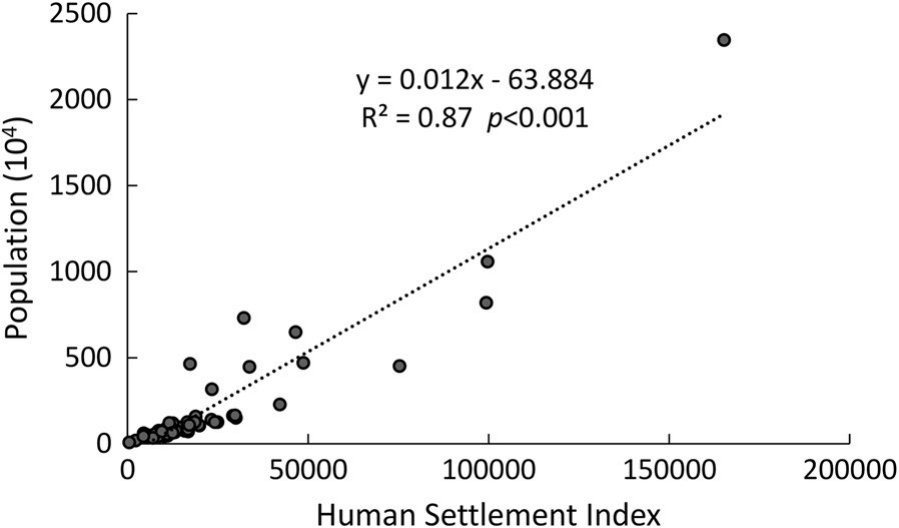
Scatterplots of the accumulated EAHSI value and population of counties of the Yangtze River Delta

**Fig. 5:**
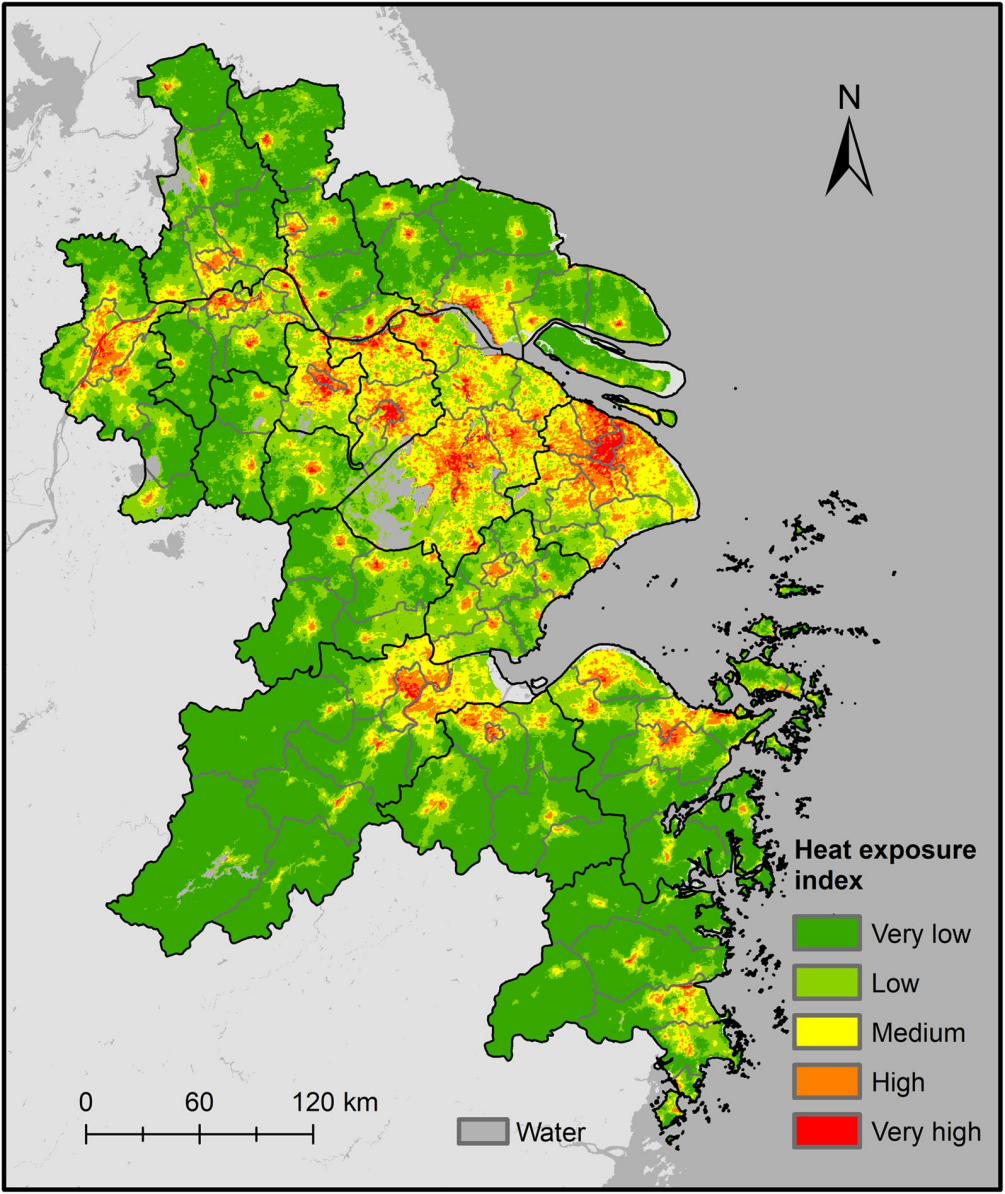
Map of the heat exposure index of the Yangtze River Delta

Figure 4 displays the scatter plot between the accumulated EAHSI and the total population at county level in 2013. The strong linear correlation, with R^2^ equal to 0.87, indicates that the EAHSI is a good proxy for the spatial delineation human exposure estimation in the YRD. The map of gridded human exposure index (Fig. 5) identifies a concentration of very high human exposure within the central areas of big cities, while moderate human exposure was found in some less-urbanized cities.

### Vulnerability

**Fig. 6:**
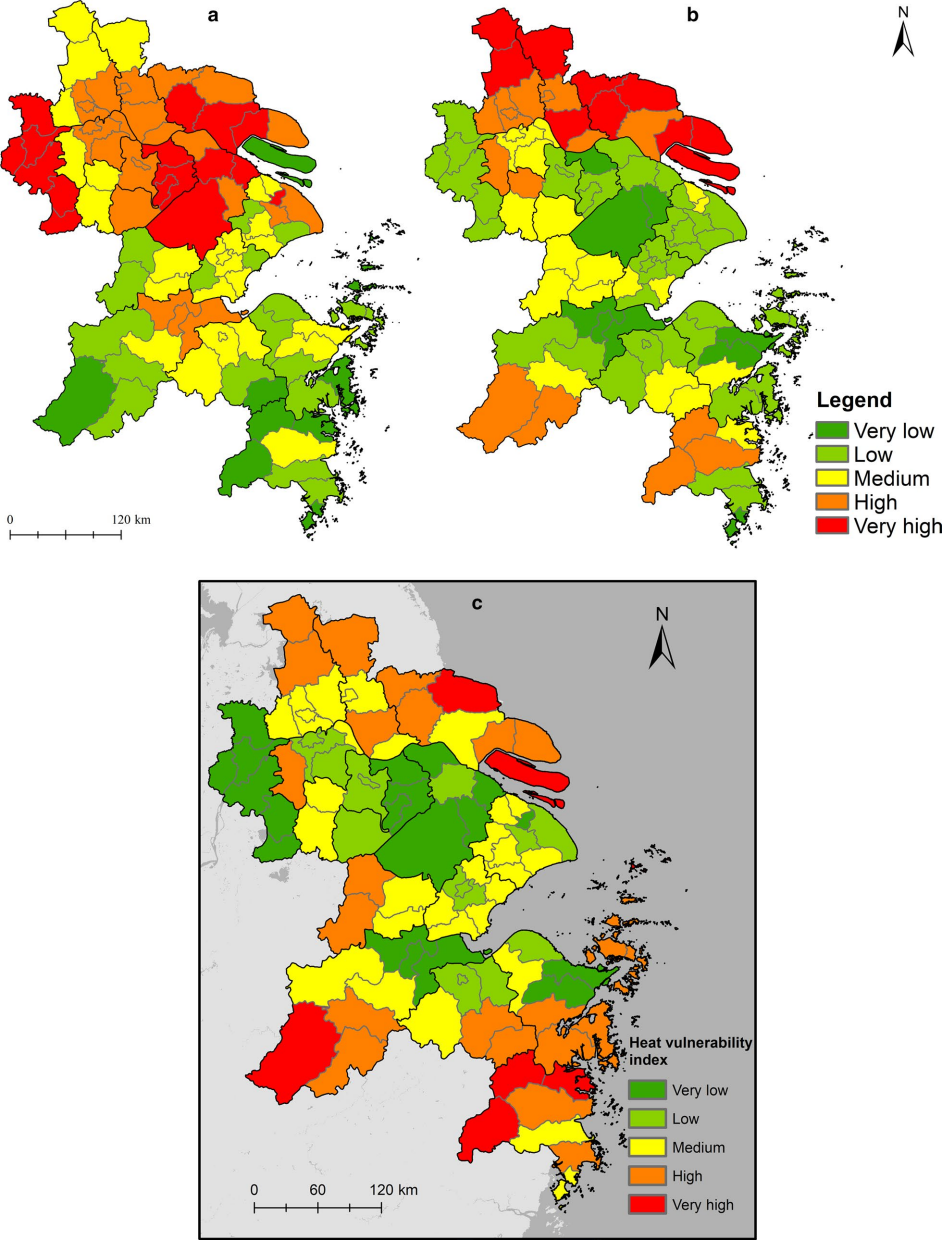
Maps of principle components **a** socioeconomic status, **b** age and **c** the heat vulnerability index of the Yangtze River Delta

As shown in Table 1, many of the six vulnerability variables were significantly correlated. Using the PCA method, the principal components with eigenvalues greater than 1.0 were used in the analysis (Table 2). These extracted factors were named based on their dominant loadings. The first factor, socioeconomic status, contributes 48.24% of the total variation among all the 6 variables. This factor is dominated by the variables that imply a high air conditioner ownership, per capita GDP and total beds of health institutions, low illiteracy rates of population (≥ 15 years), and low percentage of the elderly (≥ 60 years) living alone. Overall, the first factor identifies a group of study units with higher socioeconomic level, which contributes to a lower heat vulnerability. As depicted in Fig. 6a, areas with high socioeconomic status were mainly distributed in the most developed study units such as the downtown of Shanghai, Nanjing, Suzhou, Wuxi and Nantong. These cities were characterized by high economic conditions, good medical service access, as well as a high percentage of educated population. The second factor, age, contributes 17.12% of the total variance. It identifies a group of units with a low percentage of population over 65 years old, which contributes to a lower heat vulnerability. Areas with a high percentage of elderly population were generally scattered throughout the northeast of the study area (Fig. 6b).

**Table 1:** Spearman’s correlation values for vulnerability variables (n = 76)

	Percentage of the elderly (≥ 60 years) living alone	Percentage of population over 65 years old	Illiteracy or semi-illiteracy rates of population (≥ 15 years)	Per capita GDP (RMB Yuan)	Total beds of health institutions	Air conditioners per 100 household
Percentage of the elderly (≥ 60 years) living alone	1.00					
Percentage of population over 65 years old	*0.07*	1.00				
Illiteracy or semi-illiteracy rates of population (≥ 15 years)	0.49	*0.20*	1.00			
Per capita GDP (RMB Yuan)	− 0.39	− 0.48	− 0.40	1.00		
Total beds of health institutions	− *0.26*	− *0.19*	− 0.37	0.38	1.00	
Air conditioners per 100 household	− 0.44	− *0.28*	− 0.47	0.61	0.46	1.00

All values are statistically significant at p < 0.001 except for those in italics

**Table 2:** Principle component analysis result of social vulnerability

Components	Eigenvalue	Percentage variance explained	Variables	Loadings
(1) Socioeconomic status	2.667	48.24	Air conditioners per 100 household	0.818
			Per capita GDP (RMB Yuan)	0.801
			Illiteracy or semi-illiteracy rates of population (≥ 15 years)	− 0.715
			Percentage of the elderly (≥ 60 years) living alone	− 0.649
			Total beds of health institutions	0.641
(2) Age	1.018	17.12	Percentage of population over 65 years old	− 0.769

A composite heat vulnerability index based on the above two principle components was mapped in Fig. 6c. Very high heat vulnerability index values in the YRD were evident in the Rudong County of Nantong City, Chongming County of Shanghai, and mountainous areas in the southern YRD, which were mainly driven by the low economic and education level. In addition, vulnerability in some areas with relatively high economic level, such as the suburbs of Shanghai, were not significant but should not be ignored. Heat vulnerability in those areas was seemingly associated with a high percentage of elderly people, especially those living alone. Areas with low and very low heat vulnerability index values were generally scattered throughout the urbanized areas of Wuxi, Nanjing, Yangzhou, Hangzhou, Suzhou, Ningbo, and the downtown of Shanghai with generally high economic level, mature infrastructure, and low proportion of population sensitive to thermal risk (such as the elderly).

### Heat health risk

**Fig. 7:**
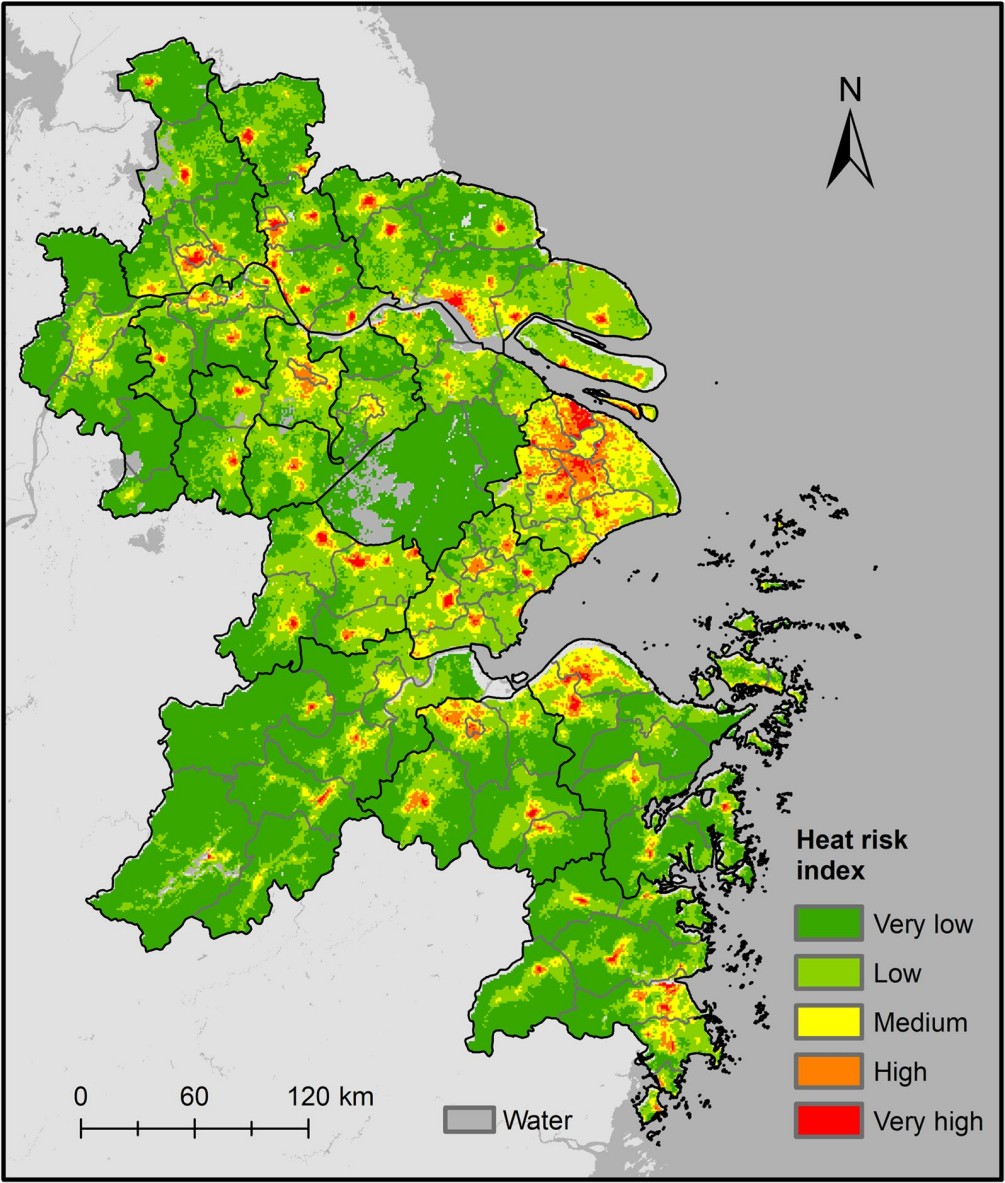
Map of the heat health risk index of the Yangtze River Delta region

The spatial pattern of heat risk index in YRD was obtained by equally weighted aggregation of three risk elements (Fig. 7). The majority of the high-risk areas are grouped together in the central urbanized areas of Changzhou, Yangzhou, Taizhou (Jiangsu Province), Jiaxing, Taizhou (Zhejiang Province), Cixi and Yuyao City of Ningbo. Notably, the heat risk index values are high in the northern area of the YRD (such as Xinghua City and Baoying County), the eastern coastal areas of Jiangsu (such as Hai’an County), and the rural areas of the southwestern study area, and this can be explained by the distribution of vulnerability index.

### Driving factors

**Fig. 8:**
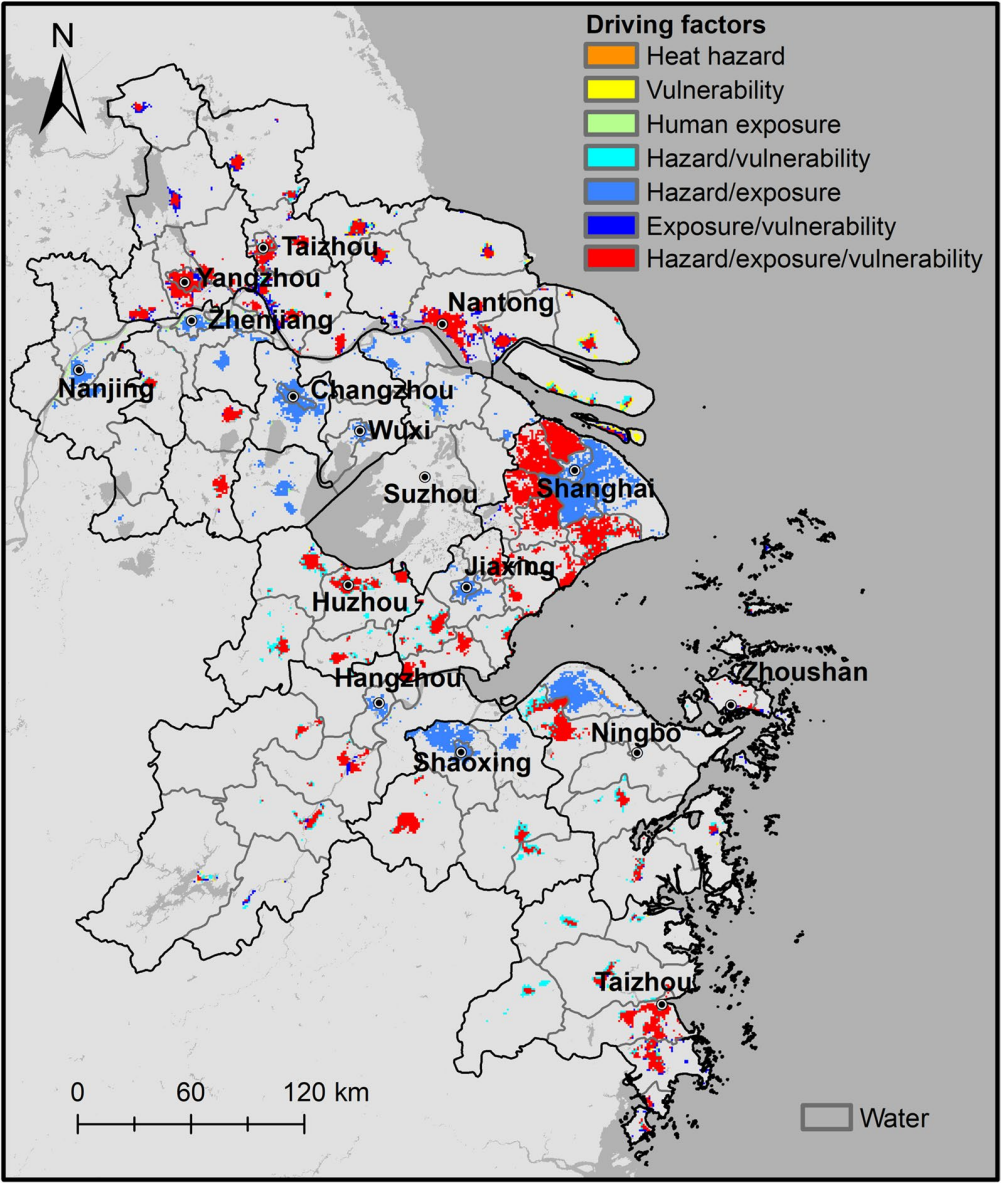
Driving factors of heat health risks in the Yangtze River Delta region

In addition to identifying the high heat health risk areas, it is also important for decision makers to recognize risk factors which play a leading role in forming these high risk areas. Here, pixels with medium or higher risk grade were considered as potential high risk areas. The heat hazard, human exposure, and heat vulnerability index were also reclassified into two grades in the same way. Then, the main driving factors that contributed to potential high heat risk areas in the YRD were identified (Fig. 8). For example, the legend “Hazard/vulnerability” in Fig. 8 means that both heat hazard and heat vulnerability grades were high whereas the heat exposure was low in the corresponding area. Heat hazard and heat vulnerability were therefore defined as the driving factors of high heat risk.

Risk areas driven by a single factor, which accounted for only 2.62% of the potential risk area, were less distributed, as depicted in Fig. 8. The heat risk patterns in 31.76% of the area were mostly driven by the distribution of heat hazard and human exposure, particularly in highly-urbanized areas of the YRD. However, in the suburbs encircling the city centers and in urban areas of some relatively small cities (e.g., suburbs of Shanghai), high vulnerability also contributes to the high risks. The heat health risks in some less-developed areas, with low human exposure index in the south and north sections of the YRD, remained high due to the assigned high heat hazard and heat vulnerability grades.

## Discussion

Previous studies have pointed out the necessity for heat health risk assessment at a finer scale [21, 23]. Given the lack of attempts of spatially explicit assessment for heat health risk, especially in developing countries, this study developed a methodology built on previous risk assessment framework and aggregating the knowledge and technologies from GIS, remote sensing and epidemiological sciences. By fully considering heat hazard, human exposure, and multidimensional vulnerability and integrating multi-sensor remote sensing images and sociodemographic data, the GIS-based methodology has been designed to be transparent and to make use of readily freely available data. The resulting pixel-level heat health risk map and the identification of driving factors can convey more information for understanding specific human risk during EHEs. It is particularly valuable in guiding local planners to develop more efficient mitigation and adaptation planning in developing countries with limited cost, time, and labour.

Previous studies pointed out that the daily minimum temperatures in the urban area were considerably higher than those in the rural area because of UHI effects [64, 65], thereby exacerbating the heat health risk on urban residents [27]. However, most spatial heat health risk studies only considered the daytime high temperature and generally omitted the impact of nighttime high temperature due to the nocturnal UHI effect. Here, cloud-free MODIS LST images were adopted to represent heat hazard during an exceptional heat wave. The nighttime LST data, which were restricted to only thermal infrared radiance from the ground, are considered a powerful proxy to more accurately represent the spatial distribution of UHI than the daytime LST [66]. Comprehensive analysis of the heat hazard in the YRD illustrated that both daytime and nighttime LSTs in urban areas were generally higher than those in rural areas. Therefore, urban residents were more likely to suffer from lasting heat stress at both day and night during EHEs, which agreed with similar work performed in other countries [13, 65, 67].

A better understanding of human exposure to EHEs required precise and spatially explicit estimation of population distribution [22, 24, 68]. In comparison with the previous studies based only on census population data, the present study conducted human exposure assessment at high spatial resolution by integrating multisource satellite images, thereby bridging the spatial mismatch with heat hazard layer. This represents a clear contribution that can lead to a better estimation of disaggregated exposure and risk at finer scales. The method for grid-based exposure assessment is characterized by the wide application of the readily available remote sensing data and flexibility of GIS technique.

It is now widely appreciated that spatial viabilities in demographic characteristics and socioeconomic status are key contributors to overall vulnerability to extreme weather events [41]. In this study, six heat vulnerability-related variables were represented by two principal components through PCA analysis to create the final heat vulnerability map. Our vulnerability map in YRD agree with the results by Chen et al. [69], which suggested that highly urbanized areas are generally much less vulnerable than rural areas. According to the PCA analysis, there exists inequity in the allocation of social resources like education opportunities and medical services between urban and rural areas. Areas with the high socioeconomic level are city districts. However, some of these places exhibit other dimensions of vulnerability. For example, Nantong City of Jiangsu Province ranks high on socioeconomic status while have a relatively high percentage of elderly people. Therefore, by separating various dimensions of vulnerability, it is possible for decision makers to understand what contributes to vulnerability and decide tailored adaptation strategies.

However, there are still some limitations that should be pointed out in this study. Firstly, the verification of the results becomes a significant gap because health and mortality records associated with previous EHEs in YRD are not available. Hospital data can be helpful in quantitative validation of heat health assessment, although the utility may be limited due to its restricted availability at temporal and spatial scales.

Secondly, we only considered the LST for the hazard analysis in this study, but the impact of EHEs on public health is in fact a function of temperature, humidity, wind speed, and other meteorological and environmental factors [15, 22]. Some studies demonstrated that the effects of air pollutants as confounders of the UHI would pose a serious threat to public health [15]; meanwhile, the “urban dry island” effect may potentially alleviate heat stress to a certain extent [70]. The synergies between temperature and the factors mentioned above should be further considered in future hazard analyses.

Thirdly, grid datasets for demographic and socioeconomic indicators are essential for vulnerability assessment but are not available at resolutions needed. In the current study, for the three risk elements, grid-based assessments on heat hazard and human exposure were conducted at a fine spatial resolution using multi-sensor remote sensing data. The spatial mismatch between exposure and hazard was therefore overcome. Still, the required grid-based datasets for other socio-economic vulnerability indicators at a finer resolution are not available for the study area. Therefore, the resulting vulnerability assessments were inevitably homogeneous within the border of the administrative units, and the spatial mismatch between vulnerability and other risk elements still exist in the current study. Furthermore, some vulnerability variables could not be considered due to the data availability. For example, although people with pre-existing illness are quite vulnerable to high temperature because of their limited mobility and self-care ability [15, 27], this important variable was not considered in this study because these data are unfortunately unavailable at the county level due to privacy. Moreover, based on the epidemiological evidence that the elderly are the most vulnerable subgroup to extreme heat and data availability [71, 72], we therefore chose the percentage of the elderly who live alone as social isolation proxy in heat vulnerability assessment following recent studies [73, 74]. Other factors such as home relocation, friends, social support, social participation, and social networks are not included because these data are not available in China census databases.

Finally, although previous literature shows the disparate contribution of heat hazard, human exposure, and heat vulnerability to human health during EHES, there are no standard weights that are widely applied [20, 41]. The identification of weightings required further knowledge about the relationships between all three elements in the specific location. Therefore, three risk elements among our heat risk index were weighted equally. Previous studies have used equal weighting with success for various factors to estimate heat health risk [21, 23, 27, 75]. Moreover, weightings can be easily modified according to new available knowledge and specific local authority requirements [23].

## Conclusion

This study presents a methodology for spatial heat risk assessment by combining freely available multi-source data, which allows for greater replicability in many other countries, especially in developing countries. Spatially, areas with higher heat hazard and human exposure are mainly concentrated in highly urbanized areas, which largely resulted in high heat health risk in the urban areas. However, the health effects of overheating during EHEs could be weakened due to low social vulnerability (associated with a low proportion of sensitive population or a high level of social and economic development) in some areas, especially Hangzhou, the central area of Shanghai, and Nanjing City. By contrast, high social vulnerability plays an important role in high heat health risk in some less-urbanized cities and in the suburban areas of mega-cities. Low-risk areas are generally found in high-altitude areas. The resultant heat health risk map is potentially applicable to decision makers when considering tailored adaptation strategies and emergency planning of heat risk.

## Abbreviations

EHEs: extreme heat events
EVI: enhanced vegetation index
DEM: digital elevation model
LST: land surface temperature
YRD: the Yangtze River Delta region
UHI: urban heat island
GIS: geographical information system
DN: digital number
MODIS: moderate-resolution imaging spectroradiometer
EAHSI: elevation-adjusted human settlement index
PCA: principal component analysis
